# Sensitivity and Uncertainty Analysis of Two Human Atrial Cardiac Cell Models Using Gaussian Process Emulators

**DOI:** 10.3389/fphys.2020.00364

**Published:** 2020-04-23

**Authors:** Sam Coveney, Richard H. Clayton

**Affiliations:** Insigneo Institute for in-silico Medicine and Department of Computer Science, University of Sheffield, Sheffield, United Kingdom

**Keywords:** cardiac electrophysiology, cell model, sensitivity analysis, uncertainty quantification, statistical model, Gaussian process

## Abstract

Biophysically detailed cardiac cell models reconstruct the action potential and calcium dynamics of cardiac myocytes. They aim to capture the biophysics of current flow through ion channels, pumps, and exchangers in the cell membrane, and are highly detailed. However, the relationship between model parameters and model outputs is difficult to establish because the models are both complex and non-linear. The consequences of uncertainty and variability in model parameters are therefore difficult to determine without undertaking large numbers of model evaluations. The aim of the present study was to demonstrate how sensitivity and uncertainty analysis using Gaussian process emulators can be used for a systematic and quantitive analysis of biophysically detailed cardiac cell models. We selected the Courtemanche and Maleckar models of the human atrial action potential for analysis because these models describe a similar set of currents, with different formulations. In our approach Gaussian processes emulate the main features of the action potential and calcium transient. The emulators were trained with a set of design data comprising samples from parameter space and corresponding model outputs, initially obtained from 300 model evaluations. Variance based sensitivity indices were calculated using the emulators, and first order and total effect indices were calculated for each combination of parameter and output. The differences between the first order and total effect indices indicated that the effect of interactions between parameters was small. A second set of emulators were then trained using a new set of design data with a subset of the model parameters with a sensitivity index of more than 0.1 (10%). This second stage analysis enabled comparison of mechanisms in the two models. The second stage sensitivity indices enabled the relationship between the L-type *Ca*^2+^ current and the action potential plateau to be quantified in each model. Our quantitative analysis predicted that changes in maximum conductance of the ultra-rapid *K*^+^ channel *I*_*Kur*_ would have opposite effects on action potential duration in the two models, and this prediction was confirmed by additional simulations. This study has demonstrated that Gaussian process emulators are an effective tool for sensitivity and uncertainty analysis of biophysically detailed cardiac cell models.

## 1. Introduction

The cardiac action potential arises from the movement of ions through channels, pumps, and exchangers in the cell membrane. At any instant, the current carried by each ionic species depends on potential difference across the cell membrane, as well as ion concentrations and the dynamics of ion channel gating. The complex interplay of currents produces depolarization and then repolarization of the membrane, which then acts to trigger release of *Ca*^2+^, initiating mechanical contraction (Fink et al., 2011).

The first model of the action potential in a cardiac myocyte was developed over 50 years ago (Noble, 1962), and since then a series of more detailed models have been developed as experimental techniques and data have improved. The present generation of models provide detailed reconstructions of the cardiac action potential (Fink et al., 2011), and computational models of cardiac cells and tissue have become valuable research tools because they can encode biophysical mechanisms into a quantitative framework, and so can be used to test and construct hypotheses (Clayton et al., 2011).

Although these detailed models are capable of simulating the behavior of real cardiac myocytes, this veracity comes at the price of complexity. Models of the cardiac action potential typically comprise a system of coupled, stiff, and non-linear ordinary differential equations. There are many model parameters and boundary conditions, which we will refer to as *model inputs* from here onward. These model inputs can be derived from experimental data, using approaches based on those pioneered by Hodgkin and Huxley in squid giant axon (Hodgkin and Huxley, 1952). However, experimental data are subject to variability and error arising from both limitations of experimental methods as well as intrinsic variability in cardiac cells. Some of these inputs, such as binding affinities and reaction rate constants, can be considered to have fixed values because they have a physical basis. However, others, such as maximum ion channel conductance, depend on the ion channel density in the cell membrane as well as other factors that are variable. These quantities may therefore vary from one cell to another, and even from beat to beat in the same cell. These considerations underlie three specific problems. First, errors and variability in data are typically not taken into account when calibrating model inputs, and taking an average of experimental data can distort model behavior (Pathmanathan et al., 2015). Second, data from different sets of experiments can result in different models of the same cell type. For example, there are several models of the human atrial action potential, all based on human data, but which show different types of behavior (Cherry and Evans, 2008; Wilhelms et al., 2012). Finally, a further complication arises from the modular nature of cardiac cell models. The equations for a particular ion channel, pump, or exchanger are often re-used in different models and so the provenance of model inputs may be very difficult to establish (Niederer et al., 2009).

Addressing these problems requires tools and approaches that can quantify how model behaviors and outputs depend on model inputs. These include sensitivity analysis, which aims to quantify the change in model output resulting from changes in one or more model inputs, and uncertainty analysis, which considers how uncertain or variable model inputs defined by a distribution or range of values influence model outputs (Saltelli et al., 2019). However, the level of detail included in the present generation of cardiac cell models means that formal sensitivity and uncertainty analysis is difficult, and so a detailed examination of how model inputs influence model behavior often relies on large numbers of numerical simulations where a different set of inputs is used for each model run (Koivumäki et al., 2014). These datasets can be used for regression analysis, which enables the sensitivity of model outputs to changes in model inputs to be assessed (Sarkar et al., 2012). Another approach is to select a set of inputs, or population of models, that produce action potentials in the range of experimental observations (Britton et al., 2013; Sánchez et al., 2014; Muszkiewicz et al., 2015). A drawback of these approaches arises from the high dimensional input space for the models; a very large number of model evaluations is needed to investigate the input space thoroughly (Clarke et al., 2008), although recent work indicates that this challenge can be overcome by constructing new models that are designed for uncertainty analysis (Pathmanathan et al., 2019).

Methods for sensitivity and uncertainty analysis using probabilistic approaches have been developed and applied in areas including climate modeling (Lee et al., 2011, 2013), and are beginning to be used for cardiac and cardiovascular flow models (Eck et al., 2016; Mirams et al., 2016). One of these approaches is to use a Gaussian process (GP) as an emulator or surrogate of a model.

A GP is a flexible non-parametric regression tool widely used for machine learning, which effectively interpolates an output surface. The GP is initially trained using a set of *design data* composed of inputs and the desired output. Once trained, a GP can be evaluated very quickly to estimate an output for a new and unseen set of inputs, and so can emulate a cardiac cell model, or simulator (Chang et al., 2015). A GP can treat uncertainty and variability explicitly, providing a probabilistic (mean and variance) estimate of the output, and so is an ideal tool for sensitivity and uncertainty analysis. It can be trained on a relatively small number of simulator runs, and so offers advantages over other approaches, such as Monte Carlo methods that require large numbers of simulator runs (Sánchez et al., 2014; Melis et al., 2017).

The aim of this study was to extend our previous work on GP emulators of cardiac cell models (Chang et al., 2015; Johnstone et al., 2015; Coveney and Clayton, 2018) to demonstrate that this approach can be used on a larger scale for a systematic sensitivity and uncertainty analysis of two biophysically detailed models of the human atrial action potential. Our objectives were (i) to undertake a comprehensive sensitivity analysis of both models, taking into account first order effects and interactions; (ii) to compare sensitivity indices calculated using emulators with those calculated using a regression technique; (iii) to identify a subset of model inputs that have the greatest influence on model outputs; and (iv) to use these inputs to compare the behavior of the two models quantitatively.

## 2. Materials and Methods

### 2.1. Human Atrial Cell Models

Several models of the human atrial action potential have been developed and are reviewed in detail elsewhere (Cherry and Evans, 2008; Grandi et al., 2011; Wilhelms et al., 2012; Sánchez et al., 2014). We selected two models for the present study, both based on data from human atrial cells. The first model was the *Courtemanche* model (Courtemanche et al., 1998). The second model was an extension of the model developed by Nygren et al. (1998), with modifications to the *I*_*Kur*_ and *I*_*to*_ currents as well as the movement of *Na*^+^ (Maleckar et al., 2009) which we refer to as the *Maleckar* model.

We chose these models because both represent the action potential of human atrial cells, and so have clinical relevance. They have been used for tissue and whole-organ scale simulations of atrial fibrillation (McDowell et al., 2011; Krogh-Madsen et al., 2012; Colman et al., 2013; Tobón et al., 2013). Furthermore, both models have a comparable set of ion channels, pumps, and exchangers, but have different representations of intracellular *Ca*^2+^ handling and different action potential shapes (Cherry and Evans, 2008).

### 2.2. Model Inputs and Outputs

The Courtemanche and Maleckar cell models include components that represent membrane electrophysiology as well as intracellular *Ca*^2+^ storage, uptake, and release. We chose to concentrate on inputs that control the maximum current density carried by ion channels, pumps, and exchangers in the cell membrane as well as those that control the rate and magnitude of uptake and release of intracellular *Ca*^2+^. We also selected the cell capacitance *C*_*m*_, and the extracellular concentrations ${\left[Na^{+}\right]}_{o}$, ${\left[K^{+}\right]}_{o}$, and ${\left[Ca^{2+}\right]}_{o}$ as additional inputs. The inputs examined in this study are listed in Table 1, where the central values given are the default for each model. We used notation for model inputs and currents as given in the original manuscripts (Courtemanche et al., 1998; Maleckar et al., 2009), and as used in the CellML implementations (http://cellml.org). For example we refer to the HERG current as *I*_*Kr*_ with maximum conductance *G*_*Kr*_, and the Kv1.5 current as *I*_*Kur*_ with maximum conductance *G*_*Kur*_.

**Table 1:** Range of inputs used for design data in each cell model.

**Input**	**Central value**	**Range**	**Units**
**COURTEMANCHE MODEL**			
*G*_*Na*_	7.8	5.85–11.70 (±50%)	nS/pF
*G*_*K*1_	0.09	0.045–0.018 (±25%)	nS/pF
*G*_*to*_	0.165	0.0826–0.0330 (±50%)	nS/pF
*fG*_*Kur*_	1.0	0.50–1.50 (±50%)	None
*G*_*Kr*_	0.0294	0.0147–0.0441 (±50%)	nS/pF
*G*_*Ks*_	0.1294	0.0647–0.1941 (±50%)	nS/pF
*G*_*Ca, L*_	0.1237	0.0619–0.1856 (±50%)	nS/pF
*G*_*b, Na*_	0.0006	0.0003–0.0010 (±50%)	nS/pF
*G*_*b, Ca*_	0.0011	0.0005–0.0017 (±50%)	nS/pF
*i*_*NaK*_*Max*	0.5993	0.2997–0.8990 (±50%)	pA/pF
*i*_*NaCa*_*Max*	1600.0	800.00–2400.0 (±50%)	pA/pF
*i*_*p, Ca*_*Max*	0.275	0.1375–0.4125 (±50%)	pA/pF
*K*_*rel*_	0.30	0.15–0.45 (±50%)	/ms
τ_*tr*_	180.0	90.0–270.0 (±50%)	ms
*i*_*up*_*Max*	0.005	0.0025–0.0075 (±50%)	mM/ms
*K*_*up*_	0.00092	0.00046–0.0014 (±50%)	mM
*C*_*m*_	100.0	75.0–125.0 (±25%)	pF
${\left[Na^{+}\right]}_{o}$	140.0	126.0–154.0 (±10%)	mM
${\left[K^{+}\right]}_{o}$	5.4	4.86–5.94 (±10%)	mM
${\left[Ca^{2+}\right]}_{o}$	1.8	1.62–1.98 (±10%)	mM
**MALECKAR MODEL**			
*P*_*Na*_	0.0018	0.0009–0.0027 (±50%)	nL/s
*G*_*K*1_	3.1	2.325–3.875 (±25%)	nS
*G*_*t*_	8.25	4.125–12.375 (±50%)	nS
*G*_*Kur*_	2.25	1.125–3.375 (±50%)	nS
*G*_*Kr*_	0.5	0.250–0.750 (±50%)	nS
*G*_*Ks*_	1.0	0.50–1.50 (±50%)	nS
*G*_*Ca, L*_	6.75	3.375–10.125 (±50%)	nS
*G*_*b, Na*_	0.0605	0.303–0.909 (±50%)	nS
*G*_*b, Ca*_	0.0590	0.0295–0.0885 (±50%)	nS
_*NaK*_*Max*	68.55	34.27–102.82 (±50%)	pA
*K*_*NaCa*_	0.0750	0.0375–0.1125 (±50%)	pA/mM^4^
*i*_*p, Ca*_*Max*	4.0	2.0–6.0 (±50%)	pA
α_*rel*_	200,000	100,000–300,000 (±50%)	pA/mM
τ_*tr*_	0.01	0.005–0.015 (±50%)	s
*i*_*up*_*Max*	28,00	1,400–4,300 (±50%)	pA
*K*_*cyca*_	0.0003	0.00015–0.00045 (±50%)	mM
*K*_*srca*_	0.5	0.25–0.75 (±50%)	mM
*K*_*xcs*_	0.4	0.2–0.6 (±50%)	Dimensionless
*C*_*m*_	50	37.5–62.5 (±25%)	pF
${\left[Na^{+}\right]}_{o}$	140.0	126.0–154.0 (±10%)	mM
${\left[K^{+}\right]}_{o}$	5.4	4.86–5.94 (±10%)	mM
${\left[Ca^{2+}\right]}_{o}$	1.8	1.62–1.98 (±10%)	mM

The rationale for this choice was that each of the selected inputs can be considered uncertain (i.e., not a physical constant), yet has a biophysical interpretation. Maximum conductances of ion channels, pumps, and exchangers depend on protein expression, and so could be expected to vary within an individual cell at different times as well as from cell to cell. Cell size and capacitance vary from cell to cell. This natural variability can be considered to be aleatoric uncertainty, which is irreducible (Mirams et al., 2016). On the other hand, the kinetics of transmembrane currents are related to ion channel biophysics, and so could be considered epistemic uncertainty, which can in principle be reduced.

Our analysis proceeded in two stages. In *Stage 1* the influence of all of the inputs listed in Table 1 was examined for a fixed pacing cycle length of 1,000 ms. In *Stage 2*, a subset of the inputs was selected on the basis of their *Stage 1* sensitivity index (see below), and a new set of emulators was built using this subset as inputs. As for *Stage 1*, the simulations in *Stage 2* were paced at a fixed pacing cycle length of 1,000 ms for 39 beats, followed by an S2 stimulus. The diastolic interval (DI) of the S2 beat in the S1–S2 pacing sequence was used as an additional input.

**Figure 1:**
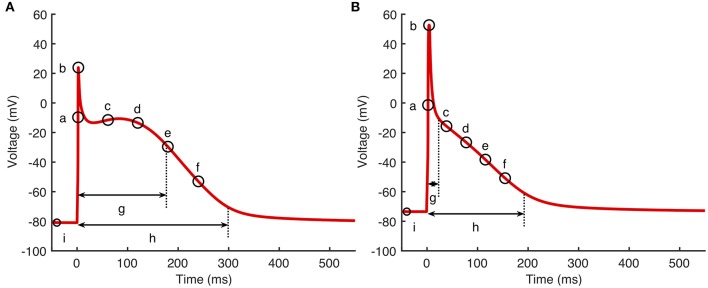
Action potential biomarkers. Nine action potential biomarkers were used as outputs to characterize each simulator run. **(A)** Courtemanche model, **(B)** Maleckar model. Biomarker labels are a: *dV*/*dt*_*max*_, b: *V*_*max*_, c–f: *V*_20_, *V*_40_, *V*_60_, and *V*_80_, g: *APD*_50_, h: *APD*_90_, i: *RestV*_*m*_. $Ca_{max}^{2+}$ and $Ca_{min}^{2+}$ not shown.

Cardiac cell models produce an output that is a time series of states. Of these, membrane voltage *V*_*m*_ and intracellular *Ca*^2+^ concentration ${\left[Ca^{2+}\right]}_{i}$ describe the time course of action potentials and *Ca*^2+^. To investigate how cell model inputs influence action potential shape, we selected nine features of the action potential that quantify its shape, based on biomarkers used in related work (Britton et al., 2013; Sánchez et al., 2014) as well as the minimum and maximum ${\left[Ca^{2+}\right]}_{i}$. These eleven outputs are shown in Figure 1 and are listed below.

- *dV*_*m*_/*dt*_*max*_—Maximum slope of the action potential upstroke.
- *V*_*max*_—Peak voltage of the action potential.
- *V*_20_, *V*_40_, *V*_60_, and *V*_80_—Membrane voltage measured at 20, 40, 60, and 80% of *APD*_90_.
- *APD*_50_ and *APD*_90_—Action potential duration at 50 and 90% of repolarization.
- *RestV*_*m*_—Resting membrane potential, calculated as the average membrane voltage over a 10 ms period, 100 ms prior to the action potential upstroke.
- $Ca_{min}^{2+}$ and $Ca_{max}^{2+}$—Minimum and maximum intracellular ${\left[Ca^{2+}\right]}_{i}$.

### 2.3. Model Implementation

Both cell models were implemented in Matlab (Mathworks Inc.), using Matlab code automatically generated from the CellML repository (http://cellml.org). The models were solved using the ode15s time adaptive solver for stiff systems of ODEs with the relative and absolute tolerance both set to 10^−6^, and the maximum time step set to 0.5 ms.

To ensure that both cell models remained stable over the range of inputs used to train the GP emulators, we made some small modifications. Previous studies (e.g., Wilhelms et al., 2012) have identified an instability in the Courtemanche model that arises from a gradual drift in the intracellular concentrations [Na^+^]_*i*_ and ${\left[K^{+}\right]}_{i}$. We therefore fixed ${\left[Na^{+}\right]}_{i}$ and ${\left[K^{+}\right]}_{i}$ at their default initial values of 11.17 and 139.00 mM, respectively in the Courtemanche model implementation. In the Maleckar model, we fixed the *I*_*K, Ach*_ current to zero.

For each run, action potentials were initiated by an inward current of 2,000 pA delivered for 2 ms in the Courtemanche model, and 750/C_*m*_ pA/pF for 6 ms in the Maleckar model. In *Stage 1*, each run comprised 40 action potentials at a cycle length of 1,000 ms, with a check to ensure that APD_90_ had reached steady state. In *Stage 2*, each run was composed of 39 S1 action potentials at a cycle length of 1000 ms and a final S2 stimulus delivered at an S1S2 interval determined by the APD_90_ of the final S1 beat, plus an offset of 10 ms, plus a diastolic interval (DI) with a range of 50–450 ms sampled using a Latin hyper-cube design with the other selected inputs as described below.

### 2.4. Gaussian Process Emulators

Our overall approach is described in detail in a previous paper (Chang et al., 2015). We treat the cardiac cell models as simulators which produce a vector of model outputs y_s_ as a function of a vector of model inputs (parameters) x such that y_s_ = *f*_*s*_(x). An emulator is then a statistical model of the simulator, sometimes known as a meta-model, a surrogate model, or a response surface model. The emulator approximates the model as y_e_ = *f*_*e*_(x), where the emulator output approximates the simulator output y_e_ ≈ y_s_ for a given input x.

In the present study we specified the emulator as a GP, where the GP hyperparameters are optimized using a set of simulator runs called *design data*. When the GP has been trained, the posterior prediction y_e_ at an input x^*^ can be evaluated, which is a probability density with an expectation and a variance. The variance for the prediction y_e_ expresses uncertainty in the prediction of the simulator behavior at x^*^ (Oakley and O'Hagan, 2002, 2004).

### 2.5. Simulator Runs for Emulator Design Data

For *Stage 1* we generated design data from 300 runs of each cell model implemented as described above. For each simulator run, a different set of inputs was generated using an optimized Latin hyper-cube design. We chose a range of variation that was a trade off between examining the effect of co-variation in model inputs while minimizing the number of model runs resulting in unphysiological behavior. We selected inputs within a range of ±50% of the central values (model defaults) given in Table 1 (i.e., from central value × 0.5 to central value × 1.5), except for *G*_*K*1_, *C*_*m*_, and extracellular ion concentrations. To reduce the incidence of unphysiological behavior (see below), these inputs had ranges of ±25, ±25, and ± 10%, respectively. Each input was scaled to vary between [0…1] over the designated range. A set of output biomarkers was obtained from the final action potential. A further set of 150 model runs were then used for emulator validation (see below). For *Stage 2*, a second set of design data was produced from 200 simulator runs of each model, with a reduced set of inputs sampled from a Latin hyper-cube as described in the results section, and other inputs set to their central value. A further set of 100 simulator runs were used for emulator validation.

**Figure 2:**
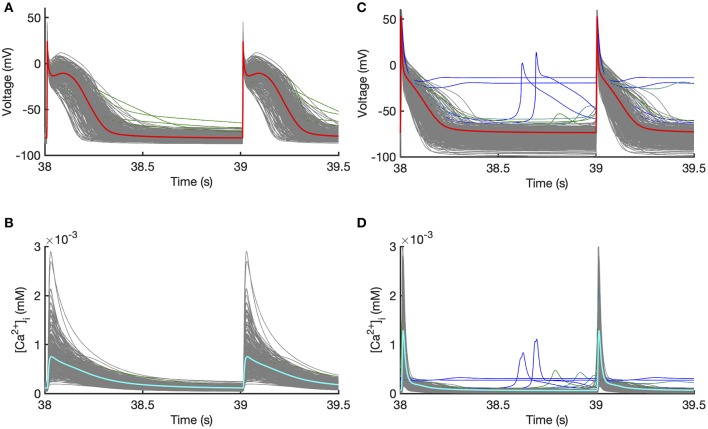
Design data. Action potentials and calcium transients produced by Latin hyper-cube sampling as described in the main text, and running each model with a pacing cycle length of 1,000 ms. Traces shown in gray were used for emulator design data, and those shown in blue were excluded (see text for details). **(A,B)** Courtemanche model. **(C,D)** Maleckar model.

Outputs from the model runs used for *Stage 1* design data in the Courtemanche model and Maleckar model are shown in Figure 2. A wide variation in action potential shapes and durations were elicited by varying the model inputs.

Model runs were removed from the design data if there was unphysiological behavior in the model outputs evidenced by pacemaking activity, a resting potential > −60 mV, *APD*_90_ >600 ms, or if *APD*_90_ of the 39th and 40th beats differed by more than 5% indicating alternans. Using these criteria, 5 out of 300 Courtemanche model runs in *Stage 1* were removed, because of a long APD or APD alternans. In the Maleckar model 14 out of 300 model runs in *Stage 1* were removed, all with pacemaking activity or failure to repolarize. In Figure 2 the removed runs are highlighted in blue. In *Stage 2* no model runs were removed for the Courtemenache model, and 5 model runs were removed for the Maleckar model.

### 2.6. GP Emulator Training

Our approach to training and using GP emulators is described in full detail elsewhere (Chang et al., 2015; Johnstone et al., 2015). Mathematical details including the expression for the posterior prediction of the emulator are provided in Supporting Information as well as in Kennedy and O'Hagan (2001); Oakley and O'Hagan (2002, 2004), and the Python code used in this study for emulator training, validation, sensitivity and uncertainty analysis is available from https://github.com/samcoveney/maGPy. Briefly, each emulator was composed of a mean function and a zero mean GP,

(1) $$\begin{array}{l} f_{e}\left(\text{x}\right)=m\left(\text{x}\right)+g\left(\text{x}\right); \end{array}$$

with a linear mean,

(2) $$\begin{array}{l} m\left(\text{x}\right)=h{\left(\text{x}\right)}^{T}\beta =\beta_{0}+\beta_{1}x_{1}+\ldots +\beta_{P}x_{P}, \end{array}$$

and a zero-mean GP,

(3) $$\begin{array}{l} g\left(\text{x}\right)\sim \mathrm{GP}(0,\sigma^{2}c(\text{x},{\text{x}}^{\prime })), \end{array}$$

where the covariance has a Radial Basis Function form,

(4) $$\begin{array}{l} c(\text{x},{\text{x}}^{\prime })=\exp\left[-\overset{P}{\underset{p=1}{\sum }}\frac{{\left(x_{p}-x_{p}^{\prime }\right)}^{2}}{\delta_{p}^{2}}\right]. \end{array}$$

In these expressions **x** = (*x*_1_, *x*_2_, …, *x*_*P*_) are the *P* inputs (parameters). The emulator hyperparameters β, δ, and σ define the emulator and need to be fitted to design data as the emulator is trained. β is a vector of length *P*+1, δ a vector of length *P*, and σ^2^ is a scalar. An optimized value for δ was obtained by maximum log-likelihood fitting to model inputs and outputs in the design data, assuming weak prior information on β and σ^2^ (Kennedy and O'Hagan, 2001), and with a fixed nugget of 10^−7^ (Andrianakis and Challenor, 2012). Mathematical details for the training and fitting procedure are given in the Supporting Information. To avoid the training process becoming trapped in a local maximum, we repeated each fit ten times, each with a different set of randomly chosen initial values for the hyperparameters. The fit with the greatest log-likelihood was then selected. We produced a separate emulator for each of the outputs shown in Figure 1.

### 2.7. Sensitivity and Uncertainty Analysis

Sensitivity and uncertainty analysis can be seen as distinct but related topics; where variance based sensitivity analysis identifies the contribution of variance in each input to variance in each output, and uncertainty analysis concentrates on estimating the uncertainty in model outputs (Saltelli et al., 2019). A GP can be evaluated for uncertain inputs **x** where each input can be either a fixed value or a probability density expressed as a mean and a variance. A GP emulator can therefore be used for variance-based sensitivity analysis.

We calculated a first order sensitivity index for each combination of input and output (Oakley and O'Hagan, 2004). For each input *w*, the first order sensitivity index describes how much the output variance would be reduced if *x*_*w*_ is fixed, while all other inputs are uncertain and are described by a mean and variance. The first order index is expressed as the ratio of variance in the emulator output when *x*_*w*_ is fixed to variance in the output when all inputs are considered uncertain.

(5) $$\begin{array}{l} S_{w}=\frac{Var\left[E\left(f_{e}\left(\text{x}|x_{w}\right)\right)\right]}{Var\left[f_{e}\left(\text{x}\right)\right]} \end{array}$$

To capture the effect of interactions between the inputs, a total effect index can be calculated. This describes the reduction in output variance when *x*_*w*_ is uncertain and all other inputs are fixed, denoted as *x*_~*w*_. It is also expressed as a proportion of the output variance when all inputs are considered uncertain.

(6) $$\begin{array}{l} S_{Tw}=\frac{Var\left[f_{e}\left(\text{x}\right)\right]-Var\left[f_{e}\left(\text{x}|x_{\sim w}\right)\right]}{Var\left[f_{e}\left(\text{x}\right)\right]} \end{array}$$

The difference between *S*_*Tw*_ and *S*_*w*_ is then the contribution of all interactions between *x*_*w*_ and *x*_~*w*_ to the variance in the output. These quantities were calculated using expressions given in the Supporting Information and described in Oakley and O'Hagan (2004).

To calculate these indices, each uncertain input was assigned a mean of 0.5 in normalized units defined by the input ranges given in Table 1. Uncertain inputs that varied ±50% were then assigned a variance of 0.02, *G*_*K*1_ and *C*_*m*_ were assigned a variance of 0.04, and the extracellular ionic concentrations were assigned a variance of 0.1.

### 2.8. Main Effects

The first order and total effect indices are both expressed as a ratio of variances, with a value in the range [0…1], and do not indicate whether an increase in output per change in input is positive or negative. To provide a sign to each index, we also calculated the main effect of each input on each output. The main effect for a single input *x*_*w*_ is the emulator output averaged over uncertain inputs, when *x*_*w*_ has a fixed value. Main effects for each combination of input and output were calculated using the procedure described in the Supporting Information, where inputs *x*_*w*_ were assigned fixed values in the range [0, 0.01…1]. The sign of the sensitivity indices was determined from the gradient of the main effect close to the central value of each input.

### 2.9. Comparison With Regression-Based Sensitivity Indices

Several recent studies have calculated sensitivity indices based on partial least squares (PLS) regression (Sobie, 2009; Sarkar and Sobie, 2010). In this approach, each model output is assumed to be a weighted sum of inputs. Thus, the model is described by the linear relationship

(7) $$\begin{array}{l} \text{y}=\text{x}\text{B}, \end{array}$$

where **y** = (*y*_1_, *y*_2_, …, *y*_*M*_) is a vector of *M* outputs, **x** = (*x*_1_, *x*_2_, …, *x*_*P*_) a vector of *P* inputs, and **B** a *P* × *M* matrix of regression coefficients. An estimate of the matrix **B**, **B_PLS_**, can be found by PLS regression on a set of design data obtained from *N* model runs, that generates an *N* × *M* matrix of inputs **Y** and an *N* × *P* matrix of outputs **X**. Each element of **X**, *x*_*i, j*_ is regularized by subtracting the mean $\bar{x_{j}}$ and dividing by the standard deviation of *x*_*j*_, and each element of **Y** is regularized in the same way. **B_PLS_** is found by minimizing the difference ||Ŷ−Ŷ||, where Ŷ = **XB_PLS_**.

The matrix **B_PLS_** can be interpreted as a matrix of sensitivity indices, provided the linear model holds. Each element of **B_PLS_**, *b*_*i, j*_ describes how changing input *x*_*i*_ results in a corresponding change in output *y*_*j*_. In both cases the change is relative to the mean value, and is a fraction of its standard deviation.

For comparison with variance based sensitivity indices, we calculated PLS sensitivity indices from the *Stage 1* design data used to train the GP emulators. The input and output matrices **X** and **Y** were constructed from regularized design data inputs and outputs. The regression matrix **B_PLS_** was then calculated using the Matlab function mvregress.

### 2.10. Emulator Validation

Each emulator was validated against an independent set of 150 simulator runs for *Stage 1* and 100 simulator runs for *Stage 2*. For each output, we calculated the mean average predicted error (MAPE) and the median individual standard error (ISE) for each validation run. The MAPE was given by

(8) $$\begin{array}{l} MAPE=\frac{100\%}{N}\frac{1}{\bar{y_{s}}}\overset{N}{\underset{n=1}{\sum }}|y_{s}^{n}-y_{e}^{n}|, \end{array}$$

where N was the number of validation runs, $y_{s}^{n}$ simulator output for run *n*, and $y_{e}^{n}$ the posterior mean emulator output for run *n*. We used the mean of the simulator output $\bar{y_{s}}$ as a denominator instead of $\left|y_{s}^{n}\right|$ to avoid bias associated with small values of $\left|y_{s}^{n}\right|$.

(9) $$\begin{array}{l} ISE=\frac{\left|y_{s}^{n}-y_{e}^{n}\right|}{\sqrt{Var\left(n,n\right)}}, \end{array}$$

where $y_{s}^{n}$ was the simulator output for run *n*, $y_{e}^{n}$ the posterior mean emulator output for run *n*, and *Var*(*n, n*) the posterior emulator variance for run *n*.

For most stage *Stage 1* and *Stage 2* emulators the MAPE was <10% and the median ISE was <1.0. Most of the differences between the output from the emulator and the output of the simulator for a given set of inputs were small, and so the fit of the emulators was considered acceptable. A table of MAPE and ISE is provided in the Supporting Information.

## 3. Results

### 3.1. *Stage 1* Sensitivity Indices

**Figure 3:**
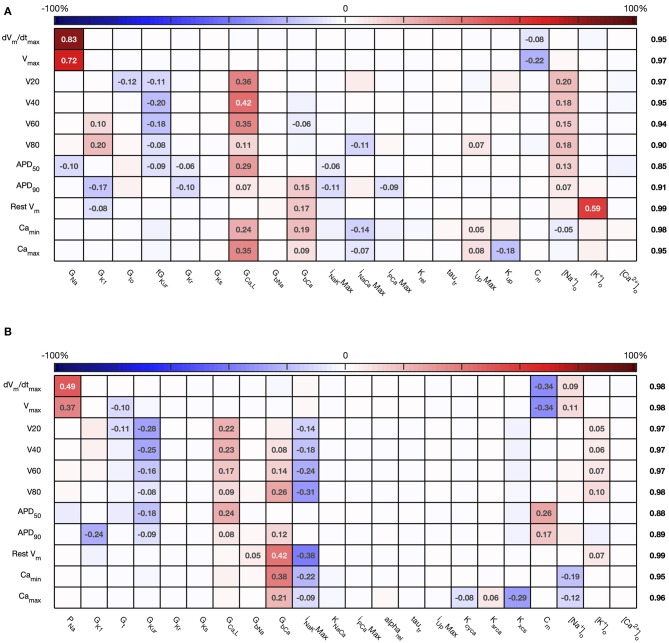
*Stage 1* first order sensitivity indices. The first order sensitivity index for each input and output is given, with a sign based on the gradient of the mean effect. Sensitivity indices <0.05 not shown, to assist visualization. The numbers at the right hand side of the table indicate the sum of the absolute values of sensitivity indices along each row. **(A)** Courtemanche model. **(B)** Maleckar model.

The first order sensitivity indices for both cell models are shown in Figure 3. Each row of the figure corresponds to one of the model outputs, and each column represents a model input. We allocated a sign to each sensitivity index based on the slope of the main effect. Main effects are described in more detail below and illustrated in **Figure 6**. The sum of the absolute values of the sensitivity indices for each output is given to the right of each grid. Since first order sensitivity indices are a ratio, a sum close to one indicates that almost all of the output variance is accounted for by the variance on each input. Smaller values for this sum, such as those for *APD*_50_ and *APD*_90_, can indicate interactions among the inputs.

**Figure 4:**
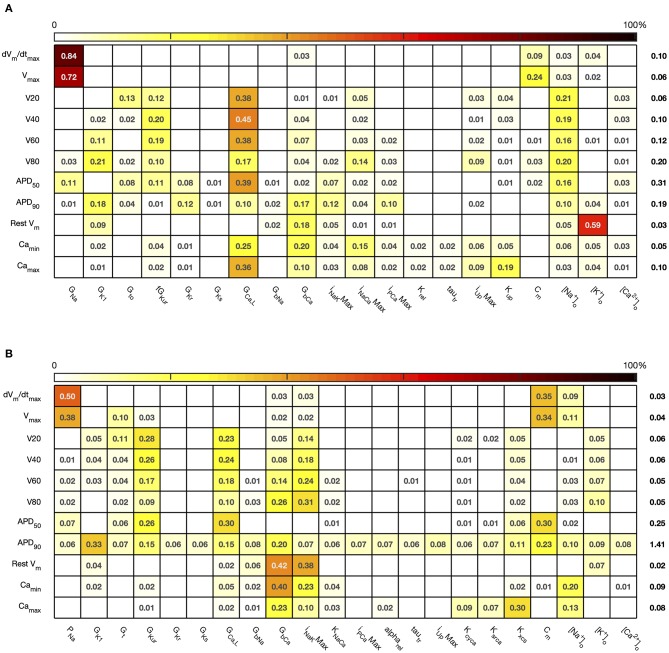
*Stage 1* total effect indices. The total effect sensitivity index for each input and output is given, indices <0.01 not shown. Numbers on right hand side are the sum across each row of the differences between the total effect index and the absolute value of the first order index. **(A)** Courtemanche model. **(B)** Maleckar model.

The total effect indices are shown in Figure 4. For each combination of input and output, the difference between the total effect index and the first order index reflects interactions with the other inputs. The sum of these differences across all of the inputs is shown at the right hand side of Figure 4. In most cases the first order and total effect indices were similar, and the sum of differences was small (≤0.12) indicating that the effect of interactions was also small. However, the sum of differences was larger for *V*_80_ in the Courtemanche model, as well as for *APD*_50_ and *APD*_90_ in both models. We conclude that in both models there are some interactions between inputs and that these interactions have an effect on repolarization. However, comparison of the sensitivity indices in Figures 3, 4 shows that these interactions appear to be distributed among all of the inputs.

**Figure 5:**
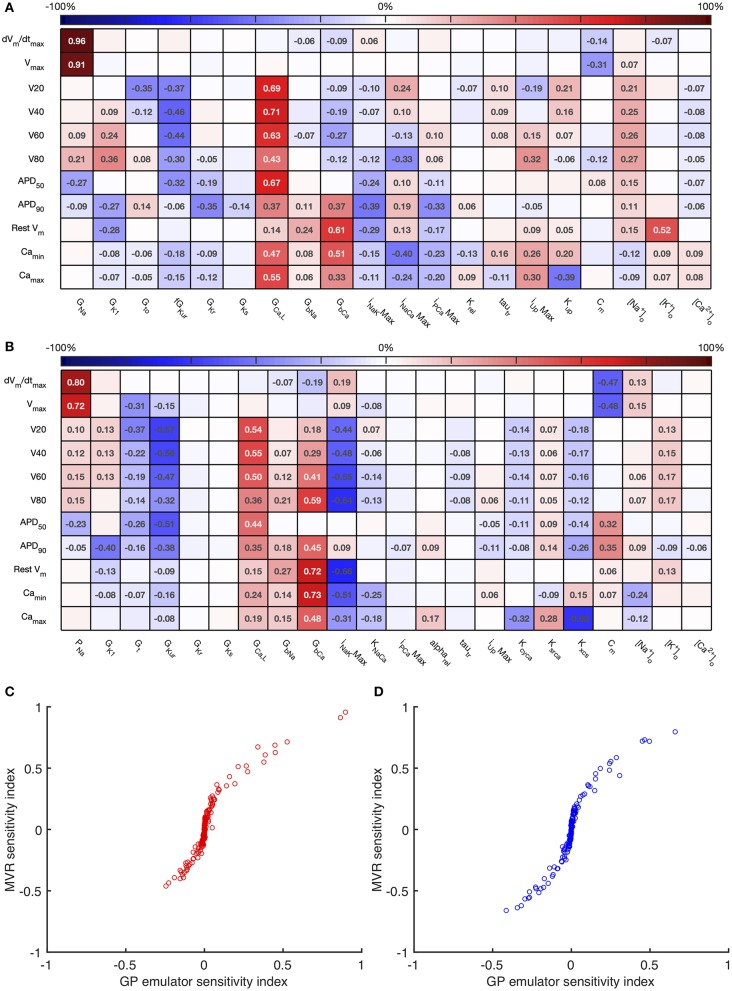
Multivariate regression sensitivity indices. The sensitivity index based on multivariate regression for each input and output is shown for the Courtemanche model **(A)** and Maleckar model **(B)**. In the lower panel, these sensitivity indices are compared with those obtained from GP emulators for the Courtemanche model **(C)**, and the Maleckar model **(D)**.

For comparison, Figure 5 shows sensitivity indices obtained by PLS regression on the emulator design data, and a comparison with variance based first order indices. The comparison plots show broad agreement, with the first order indices $S_{i}\approx B_{i}^{2}$. This relationship arises from the different definitions of *S*_*i*_ and *B*_*i*_ based on variance and standard deviation, respectively (Saltelli et al., 2012).

Overall these sensitivity indices show the contribution of uncertainty in each input to uncertainty in each output. Thus, the main contributors to uncertainty in *dV*/*dt*_*max*_ are the *Na*^+^ channel maximum conductances *G*_*Na*_ and *P*_*Na*_, and the membrane capacitance *C*_*m*_. The sign of the sensitivity indices show that these act in opposite directions, as would be expected from the role played by the *Na*^+^ current in depolarization: increasing *Na*^+^ current acts to increase *dV*/*dt*_*max*_, whereas increasing *C*_*m*_ acts to decrease *dV*/*dt*_*max*_. The bigger influence of *C*_*m*_ in the Maleckar model arises because the stimulus current density in this model scales with 1/*C*_*m*_; a larger *C*_*m*_ results in a smaller stimulus current, which in turn produces a smaller *dV*/*dt*_*max*_ and a smaller *V*_*max*_.

In both models, these sensitivity indices can be interpreted to show that increased outward currents (for example arising from increased *G*_*Kur*_) act to decrease both the voltage of the action potential plateau and action potential duration, whereas increased inward currents resulting from increased *G*_*GaL*_ and *G*_*bCa*_ have an opposite effect. This behavior is broadly what would be expected, and confirms that the sensitivity indices quantify model behavior, and can reasonably be extended to understand more complex relationships between inputs and outputs, as well as comparing behavior of the two models. In the Maleckar model *I*_*NaK*_*Max* has the opposite effect to *G*_*bCa*_. In the Courtemanche model, $Ca_{max}^{2+}$ and $Ca_{min}^{2+}$ are influenced by *G*_*CaL*_, *G*_*bCa*_, *I*_*NaCa*_*Max* and inputs that control *Ca*^2+^ handling *I*_*rel*_ and *K*_*up*_, whereas in the Maleckar model *G*_*CaL*_ and *K*_*NaCa*_ have a negligible effect, and this reflects the different formulation of *Ca*^2+^ handling in the two models (Cherry and Evans, 2008).

**Figure 6:**
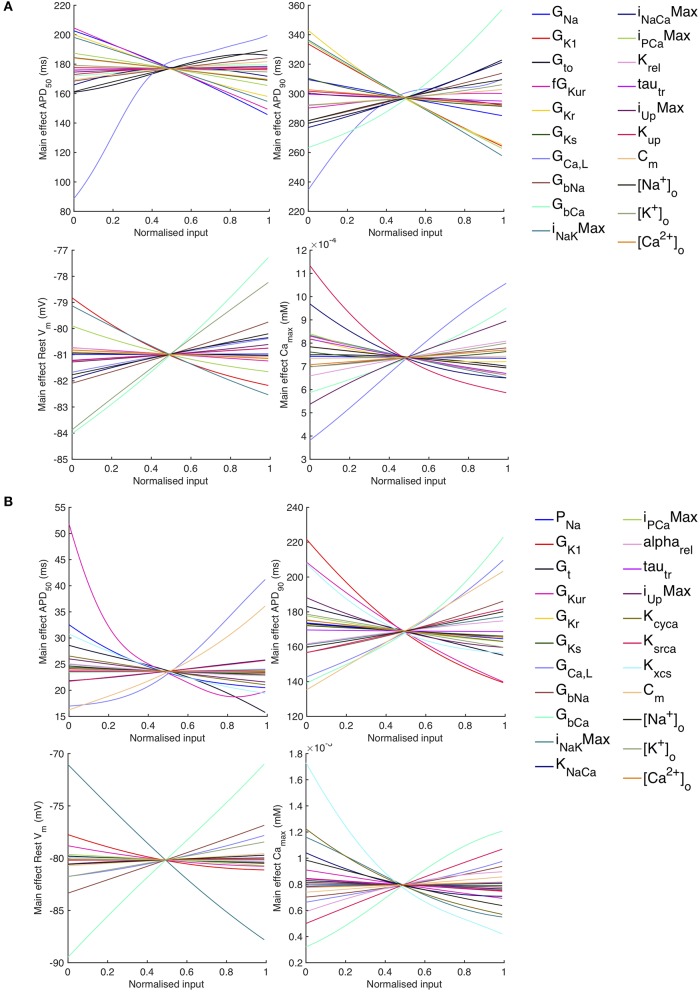
*Stage 1* selected main effects plots. Main effects for *APD*_50_, *APD*_90_, *RestV*_*m*_, and $Ca_{max}^{2+}$. **(A)** Courtemanche model. **(B)** Maleckar model.

The sensitivity analysis shows that several inputs influence *APD*_50_, *APD*_90_, *RestV*_*m*_, and $Ca_{max}^{2+}$. Figure 6 shows the main effect of each input on these outputs, for each cell model. The main effect shows the expected value of the output as each input is fixed and varied in turn across the normalized range 0…1 corresponding to the input ranges given in Table 1, while all other inputs are considered uncertain. The residual variance arising from the uncertain inputs accounts for the fact that the main effects do not converge exactly for an input value of 0.5.

Some of the main effects are comparable between the two models, for example increasing *G*_*bCa*_ acts to increase *APD*_90_, *RestV*_*m*_, and $Ca_{max}^{2+}$. Several of the effects are non-linear, for example the main effect of *G*_*CaL*_ on *APD*_50_ and *APD*_90_. However, the overall picture is complex, and it is hard to compare the different models. In order to simplify the analysis, we selected a subset of inputs for *Stage 2* of the analysis based on their sensitivity indices as described below.

### 3.2. *Stage 2* Sensitivity Analysis

For *Stage 2*, we concentrated on inputs that strongly influenced action potential shape and duration, with first order sensitivity index of more than 0.1. To simplify the analysis further, we excluded *G*_*Na*_ and *P*_*Na*_ as these inputs mainly influence action potential upstroke and amplitude. We also excluded extracellular concentrations, since these are tightly controlled in normal physiological conditions, and we excluded the inputs directly involved in the storage, uptake and release of intracellular *Ca*^2+^ because we sought to concentrate on action potential shape and duration. The *Stage 2* inputs selected for the Courtemanche model were *G*_*K*1_, *G*_*to*_, *G*_*KurMult*_, *G*_*CaL*_, *G*_*bCa*_, *I*_*NaK*_*Max*, *I*_*NaCa*_*Max*, and *I*_*PCa*_*Max*, and for the Maleckar model *G*_*K*1_, *G*_*t*_, *G*_*Kur*_, *G*_*CaL*_, *G*_*bCa*_, *I*_*NaK*_*Max*, and *C*_*m*_. All other inputs were assigned their central value from Table 1. In addition, the DI of the final action potential was introduced as an additional input to explore the dynamic behavior of the model, and this final action potential was the one that was analyzed.

**Figure 7:**
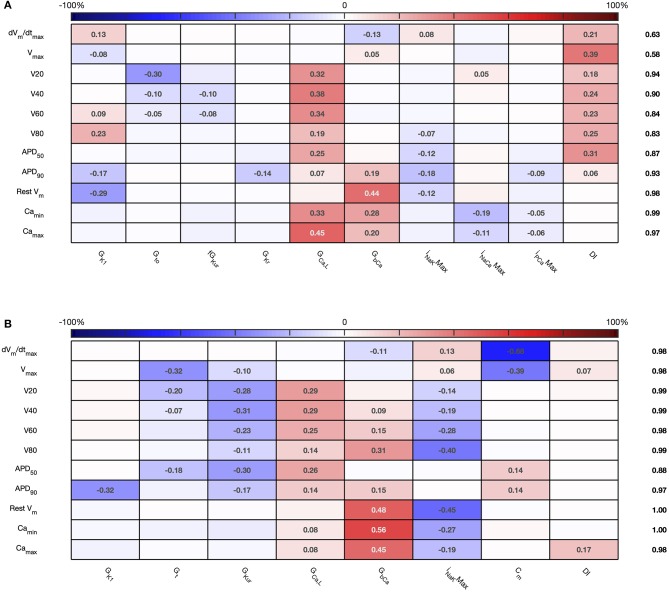
*Stage 2* first order sensitivity indices. The first order sensitivity index for each input and output is given, with a sign based on the gradient of the mean effect. Sensitivity indices <0.05 not shown. The numbers at the right hand side of the table indicate the sum of sensitivity indices along each row. **(A)** Courtemanche model. **(B)** Maleckar model.

**Figure 8:**
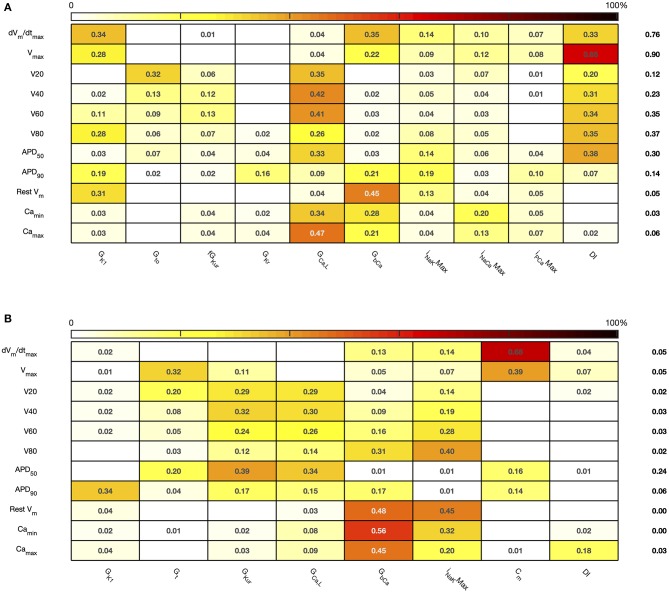
*Stage 2* total effect sensitivity indices. The total effect index for each input and output is given, indices <0.01 not shown. **(A)** Courtemanche model. **(B)** Maleckar model.

**Figure 9:**
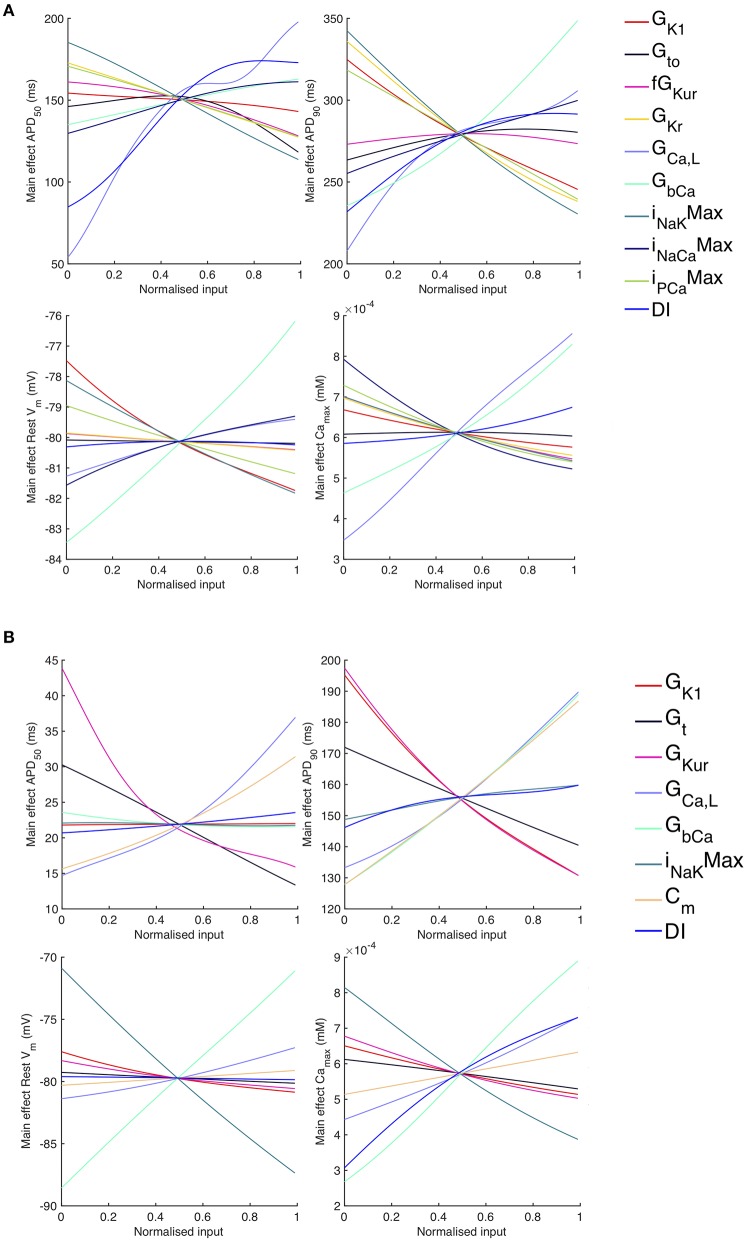
*Stage 2* selected main effects plots. *Stage 2* main effects for *APD*_50_, *APD*_90_, *RestV*_*m*_, and $Ca_{max}^{2+}$. **(A)** Courtemanche model. **(B)** Maleckar model.

The sensitivity indices for *Stage 2* are shown in Figures 7, 8, and the main effects for *APD*_50_, *APD*_90_, *RestV*_*m*_, and $Ca_{max}^{2+}$ in Figure 9.

The first order and total sensitivity indices for *Stage 2* were similar to those obtained in the *Stage 1* analysis, which is consistent with the idea that the inputs fixed for *Stage 2* had only a small effect on the outputs. *DI* was included as an additional input for *Stage 2*. In the Courtemanche model both first order and total effect indices for *DI* were larger than for the Maleckar model, indicating that *DI* has a greater influence on both the shape and duration of the action potential in the Courtemanche model compared to the Maleckar model.

The main effects plots show opposite effects of inward and outward currents on action potential duration; however *APD*_50_ as a proportion of *APD*_90_ in the Maleckar model was considerably shorter than in the Courtemanche model as a result of the different action potential shape, and so both the sensitivity indices and main effects for *APD*_50_ may not be easily comparable. This observation is reflected in the larger main effect of *DI* on *APD*_50_ in the Courtemanche model. The main effects plots also show several non-linear relationships, for example the main effect of *G*_*Kur*_ was non-linear in each of the outputs shown in Figure 9.

**Figure 10:**
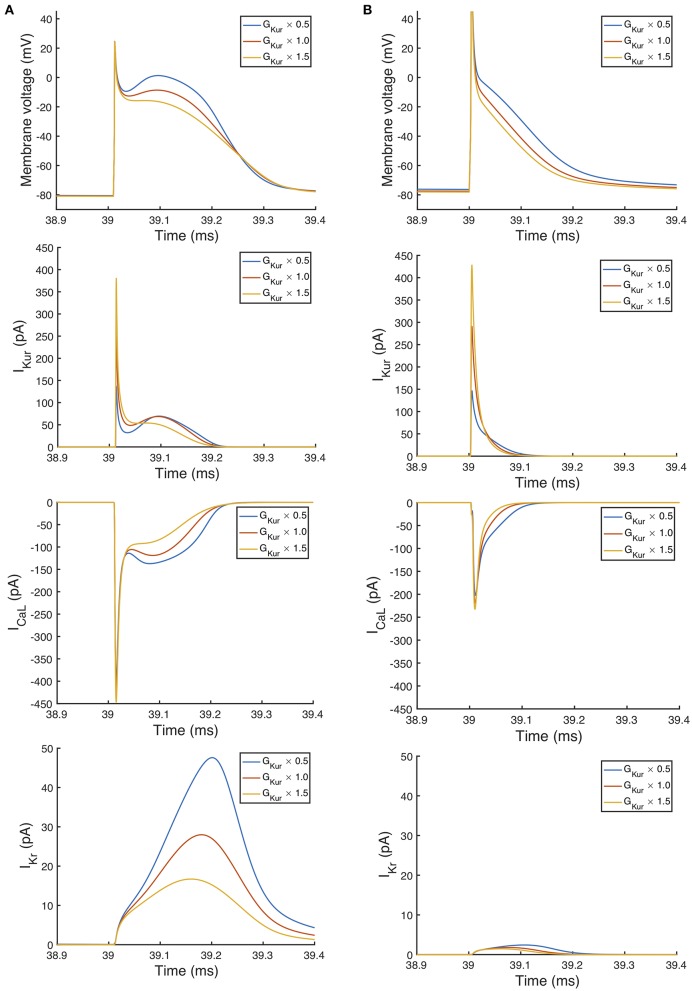
Effect of magnitude of *I*_*Kur*_ on inward and outward currents. Simulated action potential, outward current *I*_*Kur*_, inward current *I*_*Ca, L*_, and outward current *I*_*Kr*_. In each case the final beat of 40 is shown, with a pacing cycle length of 1,000 ms. **(A)** Courtemanche model. **(B)** Maleckar model.

An additional observation from Figure 9 was that the main effect of *G*_*Kur*_ on *APD*_90_ in the Maleckar model was larger, and in an opposite direction, to the main effect in the Courtemanche model. This is a prediction from the emulator analysis, and we undertook additional simulations of the cell models to verify that the emulator prediction was correct. In these additional simulations *G*_*Kur*_ was decreased to 50% and increased to 150% of its default value in both models, and each model was run for 40 beats paced at a cycle length of 1,000 ms as in the *Stage 1* analysis. The outcome of these simulations is shown in Figure 10, which shows simulated action potentials for each model, together with the principal inward and outward currents that act during the action potential plateau.

The top panels of Figure 10 show that the prediction from the emulator main effects is confirmed in these simulations: decreasing *G*_*Kur*_ results in a longer APD in the Maleckar model and a slightly shorter APD in the Courtemanche model, and *vice-versa*. Based on the simulation results shown in Figure 10, we would speculate that the mechanism by which *G*_*Kur*_ influences APD is the secondary effect of changes in action potential plateau voltage on the balance of inward and outward currents during the plateau and repolarization of the action potential, and this is consistent with earlier mechanistic studies (Greenstein et al., 2000).

Changing *G*_*Kur*_ influenced the voltage of the action potential plateau in both models, but had a different effect on the timing of repolarization. The time course of *I*_*to*_ in both models was similar, and was not strongly influenced by *G*_*Kur*_ and so is not shown. A decrease in *G*_*Kur*_ reduced the outward current during the initial part of the action potential plateau. This resulted in an increased voltage during the plateau, and a larger inward *I*_*Ca, L*_, which is voltage-dependent and acts to prolong the action potential plateau. In turn, the increased plateau voltage resulted in greater activation of the outward current *I*_*Kr*_, which is larger in the Courtemanche model compared to the Maleckar model. Thus, in the Courtemanche model, a decrease in *G*_*Kur*_ resulted in increased *I*_*Kr*_, with little change in action potential duration. In the Maleckar model *I*_*Kr*_ is much smaller, and so the increased plateau voltage did not result in increased outward current during repolarization, and so action potential duration was prolonged.

### 3.3. APD Restitution

**Figure 11:**
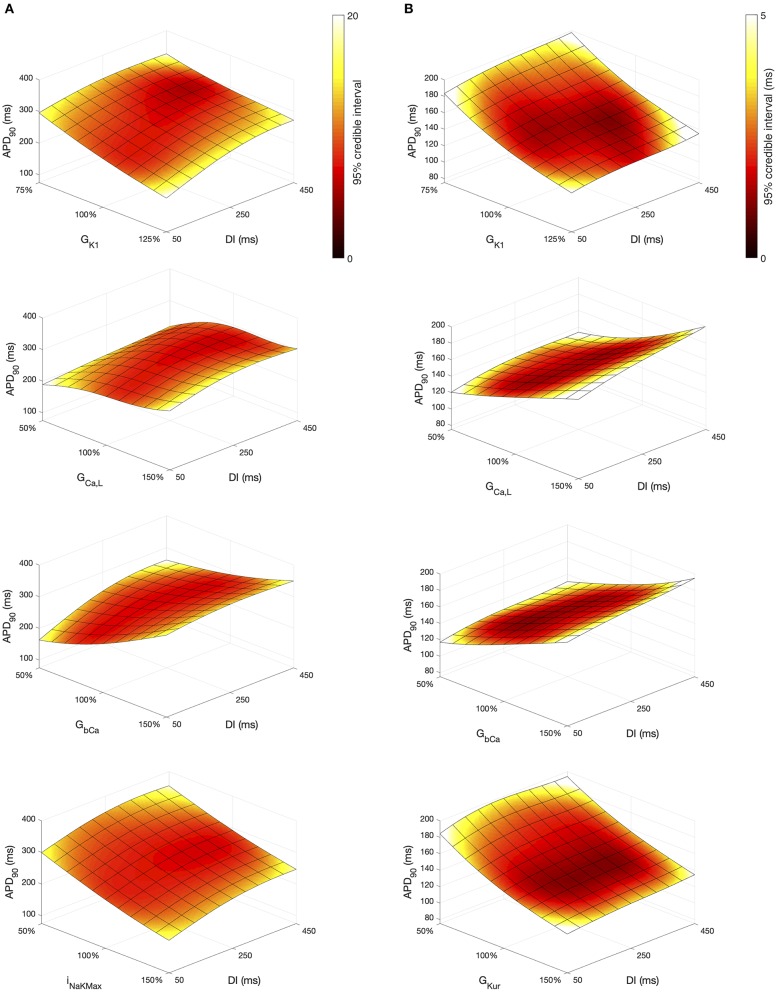
*APD*_90_ as a function of *DI* and other inputs. Each plot shows a surface of predicted mean *APD*_90_, colored by the 95% credible interval. **(A)** Courtemanche model. **(B)** Maleckar model.

The *Stage 2* analysis included diastolic interval as an input, which enabled us use the emulators to examine how different inputs affect APD restitution. In Figure 11 we have plotted a surface showing the expected value of *APD*_90_, colored by the 95% credible interval (see Supporting Information). In each of these plots the emulators were evaluated with all inputs assigned fixed values with no uncertainty, and so the 95% credible intervals reflect only uncertainty in the emulator predictions, with no uncertainty arising from uncertainty in the inputs. We assigned a value of 0.5 in normalized units to all of the inputs, except for *DI* and another inputs that were varied in each plot; these were assigned fixed values between 0 … 1.

Overall, *APD*_90_ restitution was flatter in the Maleckar model compared to the Courtemanche model, which is consistent with other studies (Cherry and Evans, 2008; Wilhelms et al., 2012). The overall effect of *G*_*K*1_, *G*_*CaL*_, and *G*_*bCa*_ on *APD*_90_ was similar in each model, with *I*_*NaK*_*Max* in the Courtemanche model having a similar effect to *G*_*Kur*_ in the Maleckar model.

However the shape of the *APD*_90_ restitution was modulated to some extent by the other inputs shown. In the Courtemanche model, decreasing *G*_*bCa*_ resulted in steepening of *APD*_90_ restitution, with a marked decrease in *APD*_90_ for short *DI* as *G*_*bCa*_ was changed from 150 to 50% of its central value. The Maleckar model also showed a marked decrease in *APD*_90_ as *G*_*bCa*_ was reduced, but this change was seen across the full range of *DI*.

### 3.4. Uncertainty Analysis

**Figure 12:**
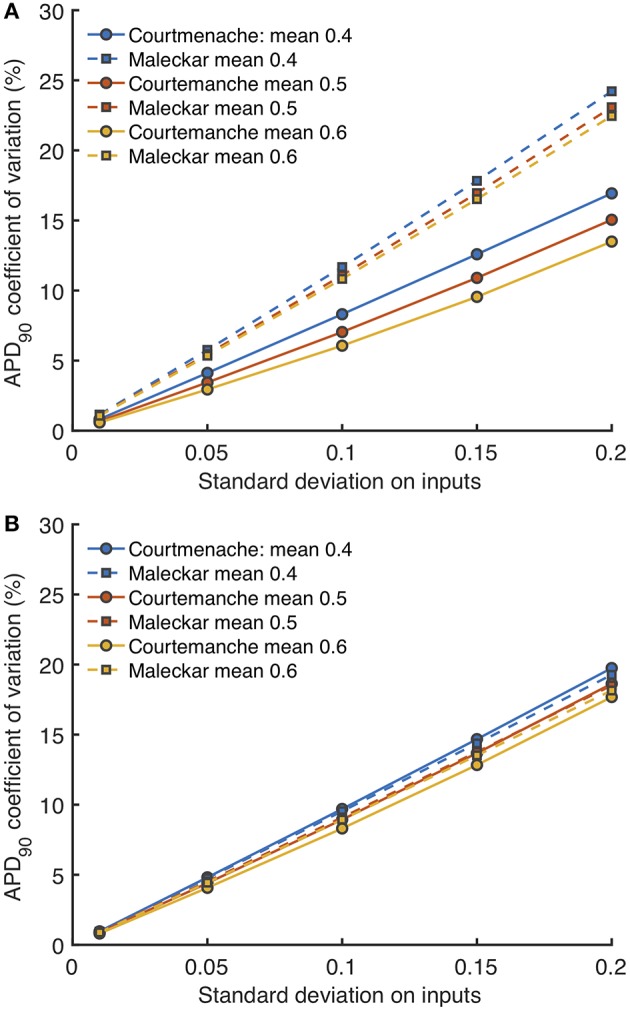
Uncertainty in *APD*_90_ and *V*_*max*_. Coefficient of variation (standard deviation divided by mean) in *APD*_90_ for *Stage 1* **(A)** and *Stage 2* plotted against imposed uncertainty on all inputs **(B)** for each model, with different mean values of 0.4, 0.5, and 0.6 in normalized units.

Uncertainty analysis aims to quantify how uncertain or variable model inputs affect uncertainty in a model output. For a cardiac cell model, uncertainty analysis enables the effects of hypothesized natural variability in model inputs to be quantified. Using the GP emulators for each cell model, we assessed how the variance in model outputs changed as variance in all of the model inputs was increased, and the results for *APD*_90_ are shown in Figure 12.

Figure 12A shows how the uncertainty in *APD*_90_ (expressed as coefficient of variation) changed as all uncertainty in all the *Stage 1* inputs (expressed as standard deviation) increased, and Figure 12B shows the effect of increasing uncertainty in all the *Stage 2* inputs. Uncertainty in *APD*_90_ increased monotonically with uncertainty in the inputs, for both *Stage 1* and *Stage 2* inputs, and in both cell models. Changing the mean value of the inputs had a small effect on output uncertainty, larger mean values reduced output uncertainty.

Reducing the number of uncertain inputs for *Stage 2* acted to reduce the output uncertainty for the Maleckar model, but increased output uncertainty slightly in the Courtemanche model. This rather unexpected finding may be due to increased interactions among the inputs, or a more problematic emulator fit. However, in the Maleckar model, the total effects for *APD*_90_ in Figure 4B are higher than the total effects for the Courtemanche model, indicating a greater degree of interaction in the Maleckar than Courtemanche models. The smaller number of inputs for *Stage 2* could then result in less uncertainty arising from interactions among uncertain inputs for the Maleckar model, and a consequent reduction in uncertainty. However, fixing some inputs for the *Stage 2* analysis may have had increased interactions in the Courtemanche model (Figure 8A), leading to an increase in uncertainty in predicted *APD*_90_.

## 4. Discussion

The main findings of this study are:

- We have demonstrated the use of GP emulators for a systematic and quantitative uncertainty and sensitivity analysis of biophysically detailed cardiac cell models.
- The first order and total effect sensitivity indices obtained using GP emulators showed both relationships that would be expected, such as the dependence of action potential upstroke on *G*_*Na*_, as well as those that were more unexpected, such as the almost negligible effect of *G*_*Ks*_ on APD. Most of the variance in model outputs was accounted for by first order sensitivities, so the interactions between the inputs considered in the present study are small.
- There was broad agreement between first order sensitivity indices obtained using GP emulators and those obtained using PLS regression.
- A subset of model inputs was identified, and behaviors predicted by the emulators were confirmed by model simulations.

We discuss these findings in more detail below, and then highlight limitations, challenges, and future directions.

### 4.1. Gaussian Process Emulation of Biophysical Models

As models of cardiac cell and tissue electrophysiology become more widely used, it is becoming increasingly important to understand how different components of the models influence model behavior, and especially how these different components interact. Biophysically detailed cardiac cell models are complex, with many interacting parts. Some of these model components may be inherited from earlier models and experiments (Niederer et al., 2009), and the process by which model parameters are fitted is also fragile when there are uncertainties associated with experimental data (Pathmanathan et al., 2015). The development and evaluation of tools for sensitivity and uncertainty analysis of cardiac models is therefore an important and growing area (Mirams et al., 2016), but much remains to be done.

The ability to evaluate emulators cheaply can be valuable for model calibration, where thorough exploration of a high dimensional input space is required (Coveney and Clayton, 2018), or if the GP emulator is used to construct outputs distributions with Monte Carlo methods. A key benefit of a GP emulator approach is the explicit handling of uncertainty. Under the assumption that inputs and outputs have Gaussian distributions, the variance on the emulator output can be calculated directly, given variances on the inputs. This enables the direct calculation of sensitivity indices, as well as enabling a systematic investigation of the way that output uncertainties depend on uncertainties in the inputs.

In this study, we have demonstrated how GP emulators can be used to systematically and quantitatively analyse biophysically detailed cardiac cell models. A GP emulator must be trained on design data. The number of simulator runs required to for design data remains an open question, and will depend on the complexity of the model output surfaces. A typical rule of thumb is to use ten times the number of inputs, and based on our previous experience (Chang et al., 2015) we opted for 300 runs for *Stage 1* and 200 runs for *Stage 2*. We also trained the *Stage 1* emulators on design data sets composed of 200 and 400 simulator runs, and obtained similar sensitivity indices to those presented here. However, further work is required to develop methods to determine the number of simulator runs needed as well as suitable metrics to determine emulator quality. Emulators should be trained on design data that fill the input space evenly, and we chose to use Latin hypercube sampling in this study (McKay et al., 1979). Other methods, such as orthogonal sampling (Bingham et al., 2009) may provide a better sampling strategy.

### 4.2. Sensitivity and Uncertainty Analysis

Recent studies have pioneered the use sensitivity indices obtained by partial least squares (PLS) regression of simulator outputs on simulator inputs, which allows a calculation of sensitivity indices (Sobie, 2009; Sarkar et al., 2012; Koivumäki et al., 2014). This approach is straightforward to implement, and we have found that it gives sensitivity indices that agree well with the square root of the first order index obtained using the GP approach (Figure 5), and the reason for this appears to be that the PLS and GP indices are based on variance and standard deviation, respectively. The overall agreement indicates that both approaches can yield similar first order sensitivity indices, although the GP easily enables calculation of interaction effects as well as first order indices. Other approaches for uncertainty and sensitivity analysis based on generalized polynomial chaos expansion have also been developed and used for analysis of cardiovascular system models (Eck et al., 2016). These approaches also enable calculation of sensitivity indices, but the relative merits of these different approaches are only beginning to be explored (Johnston et al., 2018). Both GP emulators and polynomial chaos expansions enable the explicit treatment of uncertainties, and so offer advantages for more comprehensive model analysis.

### 4.3. Human Atrial Cell Models

Models of the human atrial action potential are a subject of research interest and clinical relevance because heterogeneity in action potential shape and duration in different parts of the atria is associated with vulnerability to atrial fibrillation (Varela et al., 2016), and persistence of atrial fibrillation is associated with remodeling of the atrial action potential (Krogh-Madsen and Christini, 2012; Colman et al., 2013). There are several different models of human atrial myocytes, each with different properties (Cherry and Evans, 2008; Cherry et al., 2008; Wilhelms et al., 2012). More recent models are extensively based on data from human myocytes (Grandi et al., 2011). Most analyses of these models have focussed on the mechanisms that change action potential duration because a reduced APD increases vulnerability to atrial fibrillation, and APD can be modulated pharmacologically (Nygren et al., 1998; Zhang et al., 2005; Cherry and Evans, 2008; Koivumäki et al., 2014; Sánchez et al., 2014). These previous studies have highlighted the importance of *I*_*CaL*_, as well as *I*_*K*1_ and *I*_*Kur*_, in regulating APD. The present study adds to our understanding of the Courtemanche and Maleckar models by providing a more comprehensive view of how the model parameters affect the shape and duration of the action potential, as well as the maximum and minimum of the *Ca*^2+^ transient.

Increased inward current tends to increase amplitude of the upstroke and plateau as well as increasing APD, and increased outward current tends to have the opposite effect. The present study has highlighted how action potential duration and shape depends on the net flow of charge across the cell membrane. It is well-accepted that net current flow is finely balanced so small changes in inward and/or outward currents can influence action potential shape and duration. Our approach enables these effects to be investigated in a quantitative way. The two models examined in this study represent repolarization using a different balance of currents, and this difference is seen in the main affects plot for *APD*90 in Figure 9 and the consequences can be seen in Figure 10. The relative magnitudes of *I*_*CaL*_ and *I*_*Kr*_ in the two models are different, with much smaller *I*_*CaL*_ and *I*_*Kr*_ in the Maleckar model. This may explain why *G*_*bCa*_ exerts a stronger influence over $Ca_{min}^{2+}$ and $Ca_{max}^{2+}$ in the Maleckar model compared to the Courtemanche model.

Overall, the difference between the first order sensitivity indices (Figure 3) and the total effect indices (Figure 4) was small. The sum of these differences for each output (right hand column in Figures 4, 8) indicates more interactions in the Courtemanche model than in the Maleckar model, and that these interactions tend to affect the plateau of the action potential. These observations mean that overall the interactions between the inputs in these models do not have a strong effect on the outputs, and so we can conclude that the inputs examined in this study tend to act independently. This is a potentially important feature of the models, which could be exploited for model calibration as well as for examining mechanisms of remodeling and pharmacological action. However, it remains to be seen whether this independence is a feature of real cardiac myocytes.

### 4.4. Limitations, Challenges, and Future Directions

The use of emulators to probe detailed biophysical models is at an early stage, and so there are several limitations and challenges associated with the present study.

#### 4.4.1. Choice of Inputs

We concentrated on the effect of maximum conductances in the present study, as this reduced the complexity of the analysis. The rationale for this approach was an assumption that kinetic parameters are determined by biophysics, and so less prone to variation than the expression of ion channels, pumps, and exchangers. However, a detailed sensitivity analysis of *I*_*Kr*_ dynamics in the Courtemanche model showed that these kinetic parameters influence APD (Chang et al., 2017), and other studies have highlighted difficulties in calibrating ion channel dynamics using traditional approaches as well as showing that different formulations can have an important effect on the magnitude and time course of an ion channel current (Beattie et al., 2018). In the present study our focus was on the action potential rather than *Ca*^2+^ handling, and a detailed sensitivity analysis of the mechanisms of *Ca*^2+^ storage, release, and uptake in each model would be a valuable extension to the work presented here. So far a fully comprehensive analysis has only been done for specially constructed models (Pathmanathan et al., 2019). Nevertheless, a complete sensitivity analysis of biophysically detailed models, possibly using a hierarchical approach, remains an important challenge.

#### 4.4.2. Simulator Instability

One of the issues with a complete sensitivity analysis, highlighted in Pathmanathan et al. (2019), is that parts of the simulator input space may generate implausible behaviors. For a cardiac cell model these behaviors might be a numerical instability, spontaneous beats, or failure to repolarize. In the present study we removed simulator runs from the design data where model behavior was implausible, or where the simulator runs produced action potential alternans. We considered this to be a pragmatic approach. However, it is clearly an area for improvement because the location of these regions of input space conveys information about the model, and approaches where these locations are encoded explicitly show promise (Ghosh et al., 2018). This consideration is especially important if the inputs have a greater range of variation than those considered in the present study, to represent the effects of cellular remodeling, pathological changes, or drug action. Extending the analysis presented here to a greater range of inputs is an important future direction.

#### 4.4.3. Choice of Outputs

We selected a range of action potential features for our model outputs, these were based on measures used to describe experimental action potentials and aim to capture the main features of the action potential shape and duration. Our main focus was on the action potential. We included the maximum and minimum intracellular *Ca*^2+^ concentration as additional outputs, but did not consider the duration of the *Ca*^2+^ transient. We would not consider our choice of outputs to be definitive, and there may be better choices. A principal component analysis of the design data used in the present study showed that 95% of the output variance was accounted for by the first 6 principal components for the Courtemanche model and the first 5 for the Maleckar model. Parameterizing the action potential and *Ca*^2+^ transient so that they are described by a minimal set of features is likely to be important not only for model analysis but also for model calibration (Coveney and Clayton, 2018). Emulators that emulate time-dependent outputs have been developed, but are not yet widely used (Conti et al., 2009; Conti and O'Hagan, 2010), but could be a promising tool for extending work in this area.

#### 4.4.4. Future Directions

The aim of this study was to demonstrate the utility of sensitivity and uncertainty analysis with GP emulators. A detailed and hypothesis-driven mechanistic study was beyond the scope of the present work, but will be a valuable next step. Extending the use of emulators from models of cardiac cells to models of cardiac tissue is an important future direction, and initial studies are promising (Lawson et al., 2018). At present, tissue calculations are computationally expensive, especially for personalized meshes. However, the need to evaluate uncertainty in model predictions for use in the clinical setting requires computationally efficient approaches, and we anticipate exciting developments in this area.

## Data Availability Statement

The datasets generated for this study can be found in Clayton and Coveney (2019), Figshare Dataset—https://figshare.shef.ac.uk/articles/Design_data_for_sensitivity_analysis_of_human_atrial_myocyte_models/11309756.

## Author Contributions

RC conceived the study and undertook the analysis. SC wrote all the software used for sensitivity and uncertainty analysis. Manuscript preparation and review was shared by both the authors.

## Conflict of Interest

The authors declare that the research was conducted in the absence of any commercial or financial relationships that could be construed as a potential conflict of interest.

## Supplementary Material

**Mathematical Details:** Details of the procedures used to fit and evaluate the Gaussian process emulators, and to calculate main effects and sensitivity indices.
