# Supplementary material for: Sensitivity and Uncertainty Analysis of Two Human Atrial Cardiac Cell Models Using Gaussian Process Emulators
Source: Front Physiol. 2020 Apr 23;11:364. doi: 10.3389/fphys.2020.00364 (PMC7191317; doi:10.3389/fphys.2020.00364)
Supplement: Supplementary file 1 [file Data_Sheet_1.PDF]

Supporting information: Sensitivity and uncertainty analysis of two human atrial cardiac cell models using Gaussian process emulators

S Coveney[1,2], R H Clayton[1,2,*]

1 Insigneo Institute for in-silico Medicine, University of Sheffield, Sheffield, UK.

2 Department of Computer Science, University of Sheffield, Sheffield, UK.

* **E-mail: r.h.clayton@sheffield.ac.uk**

Introduction

In this supporting information, we include details of the mathematics that underpin the approach taken in this study. A Python implementation of the tools used is available at `https://github.com/samcoveney/maGPy`

Emulator construction

Each emulator was composed of a linear mean function and a zero mean GP,

$$f_e(\mathbf{x}) = m(\mathbf{x}) + g(\mathbf{x}). \tag{1}$$

With a linear form for the mean function $m(\mathbf{x})$ with

$$m(\mathbf{x}) = h(\mathbf{x})^T\beta, h(\mathbf{x})^T\beta = \beta_0 + \beta_1 x_1 + ... + \beta_P x_P, \tag{2}$$

and a Gaussian form for the GP covariance

$$g(\mathbf{x}) = \mathcal{GP}(0, \sigma^2 c(\mathbf{x}, \mathbf{x}') \tag{3}$$

$$c(\mathbf{x}, \mathbf{x}') = \exp\left[-\sum_{p=1}^{P}\left\{\frac{(x_p - x_p')}{\delta_p}\right\}^2\right]. \tag{4}$$

In these expressions $\mathbf{x} = (x_1, x_2, \ \dots\ , x_P)$ are P inputs (model parameters), the emulator hyperparameters β and δ are vectors of length P, and the hyperparameter σ^2 is a scalar.

Fitting emulator hyperparameters

Values for the hyperparameters were obtained by fitting to design data, assuming weak prior information on β and σ^2 [2]. In this approach, we first obtained a best estimate for δ, $\hat{\delta}$, from the maximum of the posterior log likelihood given the design data $\mathbf{D}$ and outputs $f(\mathbf{D})$, and and a prior estimate of δ, δ_0. Best estimates of β and σ^2, were then obtained from $\hat{\delta}$.

The value for $\hat{\delta}$ was chosen to be the value that optimised the posterior distribution π^*_δ, given the design data $\mathbf{D}$ and $f(\mathbf{D})$, and assuming a prior distribution of δ [2],

$$\pi^*_\delta(\delta) \propto (\hat{\sigma}^2)^{-(n-q)/2}|\mathbf{A}|^{-1/2}\,|\mathbf{H}^T\mathbf{A}\mathbf{H}|^{-1/2} \tag{5}$$

Where N was the number of design points, $Q = P + 1$, $\mathbf{H}$ an $N \times Q$ matrix given by

$$\mathbf{H} = [h(\mathbf{x}_1), h(\mathbf{x}_2),\ \dots\ , h(\mathbf{x}_N)]^T. \tag{6}$$

$\mathbf{A}$ was an $N \times N$ matrix

$$\mathbf{A} = \nu\mathbf{I} + \begin{pmatrix} 1 & c(\mathbf{x}_1,\mathbf{x}_2) & \cdots & c(\mathbf{x}_1,\mathbf{x}_N) \\ c(\mathbf{x}_2,\mathbf{x}_1) & 1 & & \vdots \\ \vdots & & \ddots & \\ c(\mathbf{x}_N,\mathbf{x}_1) & \cdots & & 1 \end{pmatrix}, \tag{7}$$

where $\nu = 10^{-7}$ was a small nugget used to reduce numerical problems with large δ [1], $\mathbf{I}$ the identity matrix, $c(.,.)$ the covariance function given by Equation 4, $\mathbf{x}_1, \mathbf{x}_2,\ \dots\ , \mathbf{x}_N$ rows of the design data $\mathbf{D}$, and

$$\hat{\sigma}^2 = \frac{1}{(N-Q-2)} f(\mathbf{D})^T \left\{ \mathbf{A}^{-1} - \mathbf{A}^{-1}\mathbf{H}\left(\mathbf{H}^T\mathbf{A}^{-1}\mathbf{H}\right)^{-1}\mathbf{H}^T\mathbf{A}^{-1} \right\} f(\mathbf{D}). \tag{8}$$

Optimised estimates of $\hat{\delta}$ were obtained by maximising the log-likelihood in equation 5 given the design data inputs and outputs, using the L-BFGS-B algorithm implemented in SciPy [1]. This approach enabled an estimate

[1]https://docs.scipy.org/doc/scipy/reference/optimize.minimize-lbfgsb.html

of $\hat{\sigma}^2$ to then be calculated from equation 8, and an estimate of $\hat{\beta}$ to be calculated from

$$\hat{\beta} = \left(\mathbf{H}^T \mathbf{A}^{-1} \mathbf{H}\right)^{-1} \mathbf{H}^T \mathbf{A}^{-1} f(\mathbf{D}), \tag{9}$$

The set of hyper parameters $\hat{\delta}, \hat{\sigma}^2$, and $\hat{\beta}$, together with the mean function given by equation 2 and the covariance function (equation 4) therefore specified each emulator, conditional on the design data inputs $\mathbf{D}$ and outputs $f(\mathbf{D})$. For a given input vector $\mathbf{x}$, the output of the emulator was then an uncertain value with a posterior mean (denoted m^*) given by

$$m^*(\mathbf{x}) = h(\mathbf{x})^T \hat{\beta} + c(\mathbf{x})^T \mathbf{A}^{-1} \left(f(\mathbf{D}) - \mathbf{H}\hat{\beta} \right), \tag{10}$$

and the posterior variance by

$$\begin{aligned} v^*(\mathbf{x}, \mathbf{x}') = \hat{\sigma}^2 \Big\{ & c(\mathbf{x}, \mathbf{x}') - c(\mathbf{x})^T \mathbf{A}^{-1} c(\mathbf{x}') + \left(h(\mathbf{x})^T - c(\mathbf{x})^T \mathbf{A}^{-1} \mathbf{H} \right) \\ & \left(\mathbf{H}^T \mathbf{A}^{-1} \mathbf{H} \right)^{-1} \left(h(\mathbf{x}')^T - c(\mathbf{x}')^T \mathbf{A}^{-1} \mathbf{H} \right)^T \Big\}. \end{aligned} \tag{11}$$

Where all the quantities are as specified above.

Uncertain emulator outputs

A powerful feature of GP emulators is that uncertainty analysis is built in, so that uncertainty on the inputs can be specified. If the probability density function of uncertain inputs is given by $\omega(\mathbf{x})$, then the posterior expectation of the emulator output with random inputs X is [3]

$$E^*[E[f_e(X)]] = \int m^*(\mathbf{x}) \omega(\mathbf{x}) \, dx, \tag{12}$$

and the variance of this expectation is

$$Var^*[E[f_e(X)]] = \iint v^*(x, x') \omega(x) \omega(x') \, dx dx'. \tag{13}$$

The expected variance of the emulator output is

$$E^*[Var[f_e(X)]] = (I_1 - Var^*[E[f_e(\mathbf{x}))]) + (I_2 - (E^*[E[f_e(\mathbf{x}))])^2) \tag{14}$$

where

$$I_1 = \int v^*(x,x)\omega(x)\,dx \tag{15}$$

and

$$I_2 = \int m^*(x)^2\omega(x)\,dx. \tag{16}$$

Our choice of linear mean, weak prior, and Gaussian correlation function for the GP enabled direct calculation of these integrals, with a further assumption that the probability density function of the inputs $\omega(\mathbf{x})$ was multivariate Gaussian with specified mean $\mathbf{m}$ (having the same size as $\mathbf{x}$) and variance $\mathbf{var}$. These expressions and their derivation are described in more detail elsewhere [2]

The expectation of the emulator output given in equation 12 can be given by

$$E^*[E[f(\mathbf{x}))] = \mathbf{R_h}^T\hat{\beta} + \mathbf{R_t}^T\mathbf{e}, \tag{17}$$

where $\mathbf{R_h}$ was a $1 \times Q$ vector, obtained from the expectation of $h(X)$ given the mean values of the uncertain inputs $\mathbf{m}$ and associated precision matrix $\mathbf{B}$, and that $h(\mathbf{x})^T$ is $(1, \mathbf{x}^T)$,

$$\mathbf{R_h} = (1, \mathbf{m}), \tag{18}$$

$\mathbf{R_t}$ is a $1 \times N$ vector, where the kth element (of N) was given by

$$R_t(k) = (1-\nu)\,|\mathbf{B}|^{1/2}\,|2\mathbf{C}+\mathbf{B}|^{-1/2}\,\exp\left(\frac{-Q_k(\mathbf{m}'_k)}{2}\right). \tag{19}$$

In this expression ν was a nugget set to 10^{-7}. $\mathbf{B}$ was the precision matrix of the input distribution $\omega(\mathbf{x})$, the inverse of a diagonal covariance matrix CV where $CV(i,j) = var(i)$ for $i = j$, and $C(i,j) = 0$ otherwise, and $var(i)$ was the ith element of the variance vector $\mathbf{var}$ giving the specified variance in input x_i. $\mathbf{C}$ was a diagonal $P \times P$ prior correlation matrix where $C(i,j) = \hat{\delta}(i)$ for $i = j$, and $C(i,j) = 0$ otherwise. The

[2] http://www.mucm.ac.uk/Pages/Downloads/Technical%20Reports/10-12%20AOH%20UAVarVar%200611.pdf

function $Q_k(\mathbf{m}'_k)$ was a scalar given by

$$Q_k(\mathbf{m}'_k) = 2\left(\mathbf{m}'_k - \mathbf{x}_k\right)^T \mathbf{C}\left(\mathbf{m}'_k - \mathbf{x_k}\right) + \left(\mathbf{m}'_k - \mathbf{m}\right)^T \mathbf{B}\left(\mathbf{m}'_k - \mathbf{m}\right) \tag{20}$$

where $\mathbf{x_k}$ was the kth row of inputs (with P elements) in the design data used to build the emulator, and $\mathbf{m}'_k$ was a $P \times 1$ vector given by

$$\mathbf{m}'_k = \left(2\mathbf{C} + \mathbf{B}\right)^{-1} \left(2\mathbf{C}\,\mathbf{x}_k + \mathbf{B}\,\mathbf{m}\right), \tag{21}$$

Finally, $\mathbf{e}$ was an $N \times 1$ vector

$$\mathbf{e} = \mathbf{A}^{-1}(f(\mathbf{D} - \mathbf{H}\hat{\beta}) \tag{22}$$

The variance of the expectation of the emulator output $Var^*[E[f(\mathbf{x}]]$ (equation 13) was given by

$$Var^*[E[f(\mathbf{x})]] = \hat{\sigma}^2\left[U - \mathbf{R}_t^T\mathbf{A}^{-1}\mathbf{R}_t + (\mathbf{R}_h - \mathbf{G}^T\mathbf{R}_t)^T\mathbf{W}(\mathbf{R}_h - \mathbf{G}^T\mathbf{R}_t)\right]. \tag{23}$$

where

$$U = (1-\nu)\,|\mathbf{B}|\,|\mathbf{B2}|^{-1/2}, \tag{24}$$

$\mathbf{B2}$ was a $2P \times 2P$ matrix,

$$\begin{aligned}
&\mathbf{B2}(1...P, 1...P) = 2\mathbf{C} + \mathbf{B},\\
&\mathbf{B2}(P+1...2P, P+1...2P) = 2\mathbf{C} + \mathbf{B},\\
&\mathbf{B2}(1...P, P+1...2P) = -2\mathbf{C},\\
&\mathbf{B2}(P+1...2P, 1...P) = -2\mathbf{C},
\end{aligned} \tag{25}$$

$\mathbf{G}$ an $N \times Q$ matrix,

$$\mathbf{G} = \mathbf{A}^{-1}\mathbf{H}, \tag{26}$$

and $\mathbf{W}$ a $Q \times Q$ matrix,

$$\mathbf{W} = \mathbf{H}^T\mathbf{A}^{-1}\mathbf{H}. \tag{27}$$

The expectation of the variance of the emulator output $E^*[Var[f(\mathbf{x}]]$ could then be calculated using equation 14, where

$$I_1 = \hat{\sigma}^2 \left[1 - trace\left(\mathbf{A}^{-1}\mathbf{R}_{tt}\right) + trace\left(\mathbf{W}\left(\mathbf{R}_{hh} - 2\mathbf{R}_{ht}\mathbf{G} + \mathbf{G}^T\mathbf{R}_t t\mathbf{G}\right)\right)\right] \tag{28}$$

and

$$I_2 = \hat{\beta}^T\mathbf{R}_{hh}\hat{\beta} + 2\hat{\beta}^T\mathbf{R}_{ht}\mathbf{e} + \mathbf{e}^T\mathbf{R}_{tt}\mathbf{e}. \tag{29}$$

In these expressions $\mathbf{R}_{tt}$ was an $N \times N$ matrix, where entry (k, l) was given by

$$R_{tt}(k,l) = (1-\nu)^2 \left|\mathbf{B}\right|^{1/2} \left|4\mathbf{C} + \mathbf{B}\right|^{-1/2} \, \exp\left(-Q_{kl}(\mathbf{m'}_{kl})/2\right), \tag{30}$$

where $\mathbf{m'}_{kl}$ was a $P \times 1$ vector

$$\mathbf{m'}_{kl} = (4\mathbf{C} + \mathbf{B})^{-1}\left(2\mathbf{C}x_k + 2\mathbf{C}x_l + \mathbf{B}\,\mathbf{m}\right), \tag{31}$$

and $Q_{kl}(\mathbf{m'}_{kl})$ a scalar given by

$$\begin{aligned} Q_{kl}(\mathbf{m'}_{kl}) = 2(\mathbf{m'}_{kl} - x_k)^T\mathbf{C}(\mathbf{m'}_{kl} - x_k) + 2(\mathbf{m'}_{kl} - x_l)^T\mathbf{C}(\mathbf{m'}_{kl} - x_l) \\ +(\mathbf{m'}_{kl} - \mathbf{m})^T\mathbf{B}(\mathbf{m'}_{kl} - \mathbf{m}). \end{aligned} \tag{32}$$

$\mathbf{R}_{ht}$ was a $Q \times N$ matrix, where the kth column was given by

$$\mathbf{R}_{ht}(k) = \mathbf{R}_t(k)\mathbf{F}, \tag{33}$$

where $\mathbf{F}$ was a $Q \times 1$ vector with entries $(1, \mathbf{m'}_k(1)...\mathbf{m'}_k(P))$, where $\mathbf{m'}_k$ was given by equation 21. Finally, $\mathbf{R}_{hh}$ was a $Q \times Q$ matrix $\mathbf{R}_h h = \mathbf{R}_h^T\mathbf{R}_h$, where $\mathbf{R}_h$ was given by equation 18.

Calculation of main effects

The main effect $M_w(x_w)$ is the emulator output averaged over uncertain inputs, when input x_w has a fixed value. The posterior expectation of $M_w(x_w)$ is a scalar, and is given by

$$M_w(x_w) = \mathbf{R_w}\hat{\beta} + \mathbf{T_w}\mathbf{e}. \tag{34}$$

This equation had the same form as equation 17. $\mathbf{R_w}$ was a $1 \times Q$ vector with $R_w(1) = 1$, and $R_w(i) = x_w$ for $i = w$, and the mean value of input i $m(i)$ otherwise for $i = 2...Q$. $\mathbf{T_w}$ was a $1 \times N$ vector, where the kth element was given by

$$T_w(k) = (1-\nu)\left\{\prod_{i \neq w}\left[\frac{B_{ii}^{1/2}}{(2C_{ii}+B_{ii})^{1/2}}\exp\left(-\frac{1}{2}\left(\frac{2C_{ii}B_{ii}}{2C_{ii}+B_{ii}}\right)(x_{i,k}-m_i)^2\right)\right]\right\} \times \exp\left(-\frac{1}{2}(x_w - x_{w,k})^T\ 2C_{ww}(x_w - x_{w,k})\right), \tag{35}$$

where $x_{i/w,k}$ was the value of the i/w^{th} (of P) input on the kth (of N) of the design data used to build the emulator, and all other quantities are as defined above. The main effects for each combination of input and output were calculated for fixed values of input x_w in the range $[0, 0.01 \ldots 1]$.

Calculation of sensitivity indices

We calculated the first order sensitivity index for input w

$$S_w = \frac{Var[E(f_e(\mathbf{x})|x_w)]}{Var[f_e(\mathbf{x})]} \tag{36}$$

and the total effect index

$$S_{Tw} = \frac{Var[f_e(\mathbf{x})] - Var[f_e(\mathbf{x})|x_{\sim w}]}{Var[f_e(\mathbf{x})]} \tag{37}$$

In each case, the denominator was calculated using the expectation given in equation 14 as described above, and the numerators were calculated as described in [3].

The numerator of equation 36 was the posterior expected value of the variance, which can be written as:

$$E^*[Var[E(f_e(\mathbf{x})|x_w]] = E^*[E[E[f_e(\mathbf{x}|x_w)]^2]] - E^*[E[f_e(\mathbf{x})]^2]. \tag{38}$$

The first term in this equation was given by

$$
\begin{aligned}
E^*[E[E[f_e(\mathbf{x})|x_w]^2]] = \hat{\sigma}^2[U_w - trace(\mathbf{A}^{-1}\mathbf{P}_w) + \\
trace\left\{\mathbf{W}\left(\mathbf{Q}_w - \mathbf{S}_w\mathbf{A}^{-1}\mathbf{H} - \mathbf{H}^T\mathbf{A}^{-1}\mathbf{S}_w^T + \mathbf{H}^T\mathbf{A}^{-1}\mathbf{P}_w\mathbf{A}^{-1}\mathbf{H}\right)\right\}] + \\
\mathbf{e}^T\mathbf{P}_w\mathbf{e} + 2\hat{\beta}^T\mathbf{S}_w\mathbf{e} + \hat{\beta}^T\mathbf{Q}_w\hat{\beta}, \qquad (39)
\end{aligned}
$$

where U_w was a scalar

$$
U_w = (1-\nu)\prod_{i\neq w}\left(\frac{B_{ii}}{B_{ii}+4C_{ii}}\right)^{1/2}, \qquad (40)
$$

$\mathbf{Q}_w$ a $Q \times Q$ matrix, which was assembled in the following steps

$$
\begin{aligned}
&Q_w(1,1) = 1 \\
&Q_w(1,2...Q) = \mathbf{m}(1...P) \\
&Q_w(2...Q,1) = \mathbf{m}(1...P) \\
&Q_w(2...Q,2...Q) = \mathbf{m}\,\mathbf{m}^T \\
&Q_w(w+1,w+1) = Q_w(w+1,w+1) + \mathbf{B}_{ww}^{-1}, \qquad (41)
\end{aligned}
$$

$\mathbf{S}_w$ a $Q \times N$ matrix, where the $(k,l)^{th}$ element was

$$
S_w(k,l) = (1-\nu)\,E[h_k(x)]\prod_{1\leq i\leq P}\left(\frac{B_{ii}^{1/2}}{(2C_{ii}B_{ii})^{1/2}}\right)\exp\left[-\frac{1}{2}\left(\frac{2C_{ii}B_{ii}}{2C_{ii}+B_{ii}}(x_{i,l}-m_i)^2\right)\right] \qquad (42)
$$

with

$$
E[h_k(x)] = \begin{cases} 1 & \text{if } k=1 \\ m_k & \text{if } k\neq w \\ \frac{2C_{kk}x_{k,l}+B_{kk}m_k}{2C_{kk}+B_{kk}} & \text{if } k=w \end{cases}, \qquad (43)
$$

$\mathbf{P}_w$ an $N \times N$ matrix, where the $(k,l)^{th}$ element was

$$P_w(k,l) = (1-\nu)^2 \left\{ \prod_{i \neq w} \left[\frac{B_{ii}}{2C_{ii}+B_{ii}} \exp\left(-\frac{1}{2} \left(\frac{2C_{ii}B_{ii}}{2C_{ii}+B_{ii}} \right) \left[(x_{i,k}-m_i)^2 + x_{i,l}-m_i)^2 \right] \right) \right] \right\}$$
$$\times \left\{ \left(\frac{B_{ww}}{4C_{ww}+B_{ww}} \right)^{1/2} \exp\left(-\frac{1}{2} \left(\frac{1}{4C_{ww}+B_{ww}} \right) \left[4C_{ww}^2 (x_{w,k}-x_{w,l})^2 + 2C_{ww}B_{ww} \left((x_{w,k}-m_w)^2 + (x_{w,l}-m_w)^2 \right) \right] \right) \right\} \qquad (44)$$

The second term was given by

$$E^*[E[f_e(\mathbf{x})]^2] = \hat{\sigma}^2 \left[\mathbf{u} - \mathbf{T}\mathbf{A}^{-1}\mathbf{T}^T + \left\{ \mathbf{R} = \mathbf{T}\mathbf{A}^{-1}\mathbf{H} \right\} \mathbf{W} \left\{ \mathbf{R} = \mathbf{T}\mathbf{A}^{-1}\mathbf{H} \right\}^T \right] + \left(\mathbf{R}\hat{\beta} + \mathbf{T}\,\mathbf{e} \right)^2 \qquad (45)$$

The first term of the numerator in equation 37 was the posterior expected value of the variance, calculated using equation 14. The second term was written as

$$E^*[Var(f_e(\mathbf{x})|x_{\sim w})] = E^*[E[E[f(\mathbf{x})|x_{\sim w}]^2]] - E^*[E[f(\mathbf{x})]^2], \qquad (46)$$

with the first part calculated using equation 39 with the set x_w replaced with the set $x_{\sim w}$, and the second term calculated using equation 45.

Emulator validation

Table 1: **Mean average prediction error (MAPE) for each emulator (%).**

	Courtemanche		Maleckar	
Output	Stage 1	Stage 2	Stage 1	Stage 2
dV/dt_{max}	3.03	2.50	1.82	1.15
$V_{amplitude}$	2.50	3.52	1.10	0.85
V_{20}	10.84	7.30	5.99	2.07
V_{40}	11.50	10.31	3.67	1.61
V_{60}	7.55	6.60	2.00	1.29
V_{80}	2.03	2.11	1.25	0.68
APD_{50}	8.50	11.61	10.12	6.75
APD_{90}	4.61	5.22	6.04	2.02
$RestV$	0.23	0.21	0.34	1.07
Ca^{2+}_{min}	2.84	2.02	5.54	4.87
Ca^{2+}_{max}	5.92	2.86	10.50	2.16

References

[1] Ioannis Andrianakis and Peter G. Challenor. The effect of the nugget on Gaussian process emulators of computer models. *Computational Statistics and Data Analysis*, 56(12):4215–4228, 2012.

[2] Marc C. Kennedy and Anthony O'Hagan. Bayesian calibration of computer models. *Journal of the Royal Statistical Society: Series B (Statistical Methodology)*, 63(3):425–464, aug 2001.

[3] Jeremy E. Oakley and Anthony O'Hagan. Probabilistic sensitivity analysis of complex models: a Bayesian approach. *Journal of the Royal Statistical Society: Series B (Statistical Methodology)*, 66(3):751–769, aug 2004.
